# Colorectal Cancer in Younger and Elderly Patients: A Real-World Analysis from the Modena Cancer Center

**DOI:** 10.3390/cancers18183063

**Published:** 2026-09-21

**Authors:** Bianca Medici, Andrea Spallanzani, Annalisa Fontana, Massimiliano Salati, Salvatore Natalizio, Alberto Bertolotti, Cristina Petrangelo, Flavia Siciliano, Elisa Pettorelli, Riccardo Cuoghi Costantini, Massimo Dominici, Fabio Gelsomino

**Affiliations:** 1 Division of Medical Oncology, Department of Medical and Surgical Sciences for Children and Adults, University Hospital of Modena, 41125 Modena, Italy; 2 Department of Training, Research and Innovation, University Hospital of Modena, 41125 Modena, Italy; 3 Unit of Clinical Statistics, University Hospital of Modena, 41125 Modena, Italy

**Keywords:** early-onset, colorectal cancer, clinicopathological features

## Abstract

**Background:** Early-onset colorectal cancer represents a growing global health challenge. Although differences between young and elderly patients are recognized, direct comparison of extreme age groups may further characterize their distinct clinical and therapeutic profiles. **Methods:** This is a retrospective, single-centre, observational study, comparing young patients (Group A: ≤50 years, n = 153) with elderly patients (Group B: ≥75 years, n = 533) during the period 1990–2025. **Results:** Group A presented higher rates of stage IV presentation (66.9% vs. 47.1%, *p* < 0.001), T4 tumors, N2 nodal involvement, distal tumor localization and synchronous metastasis (87.5% vs. 70.6%, *p* = 0.001), predominantly involving the liver and peritoneum. Younger patients received more intensive adjuvant and metastatic systemic treatment. The mOS was 52.8 months for Group A compared to 26.4 months for Group B (HR 2.29, 95% CI 1.57–3.54, *p* < 0.001) and DFS was longer in Group A (28.6 vs. 19.1 months; HR 2.26, 95% CI 1.55–3.29, *p* < 0.001). No statistically significant differences were observed in first- or second-line PFS between the cohorts. **Conclusions:** Early-onset colorectal cancer exhibits a more adverse clinicopathological profile. Younger patients showed longer overall and disease-free survival in this cohort, although these findings should be interpreted with caution given potential differences in treatment exposure, baseline fitness, comorbidity burden and the substantial temporal heterogeneity of the study period.

## Simple Summary

Colorectal cancer in young adults is an alarming global challenge, yet its underlying biology remains poorly understood. Bridging this knowledge gap, this study analyzes a group of 686 patients across thirty-five years, directly comparing extreme age cohorts: adults aged fifty or younger and elderly patients aged seventy-five or older. The study identifies several clinical, pathological and molecular features associated with colorectal disease in younger and elderly patients. By unraveling the complex interplay between adverse clinicopathological features and treatment intensity, this study provides real-world evidence to refine risk assessment and tailor therapies for younger populations.

## 1. Introduction

Early-onset colorectal cancer (EOCRC), generally defined as colorectal cancer (CRC) diagnosed in individuals aged 50 years or younger, has emerged as a significant and alarming global public health challenge [1,2,3,4,5].

While the overall incidence and mortality of CRC have steadily declined in older populations over the past few decades, largely due to the success of screening programs and improved disease management, the trend is strikingly opposite in younger adults [1,5,6,7,8,9]. This epidemiological shift has attracted increasing attention because it is occurring in populations that have traditionally been considered at relatively low risk for CRC and therefore have historically not been targeted by routine screening strategies. The progressive increase in disease incidence among younger adults has consequently raised important questions regarding the adequacy of current prevention and early-detection approaches.

Current data indicate that EOCRC now accounts for approximately 10% of all new CRC diagnoses, with projections suggesting that by 2030, 1 in 10 colon cancers and 1 in 4 rectal cancers will be diagnosed in patients aged 50 years or younger [3,10,11].

The clinical profile of EOCRC is markedly distinct from later-onset disease. Younger patients are more likely to be diagnosed at advanced stages (Stage III or IV) and frequently present with more adverse histopathological features, such as poorly differentiated cells, mucinous components and signet ring cell morphology. Furthermore, EOCRC shows a strong predilection for the distal colon and rectum, often manifesting with symptoms like rectal bleeding and abdominal pain. These symptoms are frequently underestimated or misattributed to benign conditions, leading to significant diagnostic delays ranging from several months to over a year [11,12,13,14]. The combination of delayed diagnosis and potentially more adverse disease characteristics may influence clinical outcomes, although the prognostic differences between EOCRC and later-onset CRC remain an area of ongoing investigation. In addition, the clinical consequences of a CRC diagnosis at a young age extend beyond cancer-related outcomes, potentially affecting professional activity, family responsibilities, fertility and long-term quality of life.

From a biological and molecular perspective, EOCRC is a heterogeneous disease. Approximately 16% to 25% of cases may harbor pathogenic germline variants associated with hereditary cancer predisposition syndromes, most notably Lynch syndrome, whereas most cases are sporadic and lack an identifiable hereditary cause. The rising incidence in subsequent birth cohorts suggests that early-life exposures to modifiable risk factors, including Westernized diets, obesity, sedentary behavior, antibiotic use and alterations in the gut microbiome, play a critical role in its pathogenesis [1,2,9,11,12,15]. However, the contribution of these factors is likely complex and multifactorial and no single exposure has been identified as sufficient to explain the observed epidemiological trends.

Despite the growing prevalence of this condition, many aspects of its unique biology and the factors driving the shift toward younger age groups remain poorly understood. Understanding the clinicopathological and molecular disparities between young and older patients may inform ongoing discussions on CRC screening strategies, improving early detection and tailoring therapeutic strategies [7,11,12,15,16,17,18]. At the same time, comparisons between younger and older patients are important for determining whether differences in stage at diagnosis, tumor characteristics, treatment patterns, and survival are intrinsically related to age or may instead reflect differences in clinical presentation and disease management. Such comparative analyses may help identify clinically relevant features that could inform risk stratification and individualized approaches to CRC prevention and treatment.

Although differences between younger and older patients with CRC have been widely described, a comprehensive comparison of patients at the extremes of the age spectrum integrating clinicopathological, molecular, treatment, and survival characteristics remains clinically relevant. We therefore compared patients aged ≤50 years, representing early-onset CRC, with patients aged ≥75 years to provide an integrated real-world characterization of these distinct populations.

## 2. Materials and Methods

### 2.1. Study Design and Patient Population

This exploratory, retrospective, single-center study was conducted at the Modena Cancer Center to investigate the clinical and pathological differences between younger and elderly patients with colorectal cancer.

The study population was identified from a comprehensive institutional database including 2345 patients diagnosed with colorectal cancer (CRC) between 1990 and 2025, covering stages I to IV and an age range from 27 to 100 years. Patients were eligible for inclusion if they were aged ≥18 years and had a histologically confirmed diagnosis of stage I–IV CRC (adenocarcinoma histology), with hospital and/or outpatient medical records available for the collection of the relevant clinical information.

For the purpose of the present comparative analysis, patients were selected according to two predefined age groups: Group A, including younger patients aged ≤50 years (therefore, patients aged exactly 50 years were included in the EOCRC group), and Group B, including elderly patients aged ≥75 years.

Patients aged 51–74 years were excluded to allow a focused comparison between the two predefined extreme age groups.

Among patients belonging to the two predefined age groups, those with insufficient clinical documentation to allow reliable assessment of the study variables were excluded from the final comparative population. Missing data may reflect the unavailability of older medical records and fragmented care pathways, with some patients receiving subsequent treatment or follow-up at other healthcare facilities.

**Figure 1:**
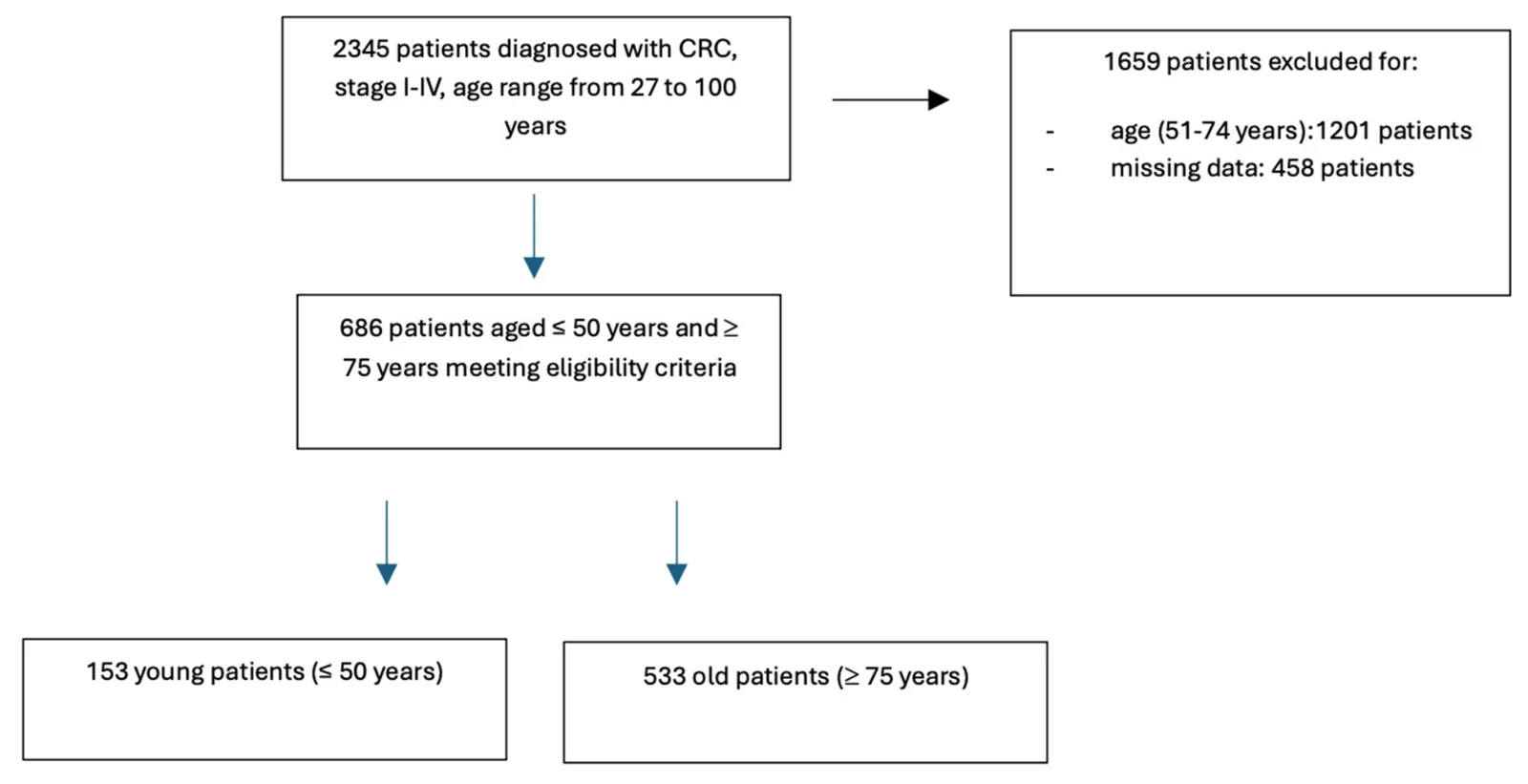
Diagram showing the population selection process.

Following this selection process, 686 patients were included in the final analysis (Figure 1).

The study evaluated demographic characteristics, comorbidities and risk factors, clinical presentation, pathological characteristics, molecular profile, treatment patterns in localized and metastatic disease and clinical outcomes.

Because of the retrospective design and the long study period, the availability of individual variables varied across patients. Analyses of individual variables were therefore performed using the available cases, with patients with missing information for a specific variable excluded from that particular analysis.

### 2.2. Data Collection and Variables

The analysis included a broad range of variables, such as:

- Patient demographics, presence of comorbidities (including cardiovascular, autoimmune, renal diseases) and family history or Lynch syndrome;
- Known lifestyle risk factors such as smoking, alcohol consumption, and obesity. Data were evaluated as a composite binary variable (presence vs. absence). Patients were classified as having a risk factor if they presented with at least one of the following criteria at baseline: (i) current or former smoking history, (ii) active or past habitual alcohol consumption, or (iii) obesity, defined as a Body Mass Index (BMI) ≥ 30 kg/m^2^. Patients without any of these features were classified as having no risk factors;
- Clinical presentation at diagnosis, specifically symptoms like a change in bowel habits, abdominal pain and anemia;
- Pathological assessment focused on tumor characteristics including TNM stage, grading and anatomical location (left colon, right colon or transverse);
- Mutational profile evaluated for KRAS, NRAS, and BRAF V600E status, as well as mismatch repair (MMR) proficiency or deficiency. HER2 and other emerging molecular alterations were not systematically available across the study period and were therefore not included in the present analysis;
- Treatment patterns for both locally advanced and metastatic settings, including surgical intervention, adjuvant chemotherapy schedules (e.g., XELOX, FOLFOX, or capecitabine), and the number of treatment lines received in the metastatic stage;
- Survival outcomes, including overall survival (OS), disease-free survival (DFS), and progression-free survival (PFS).

### 2.3. Statistical Analysis

Statistical analysis was performed to compare clinical, pathological, molecular, and treatment characteristics between the two age groups. Categorical variables were summarized as absolute frequencies and percentages. Logistic regression models were used to estimate odds ratios (ORs), when adequate. For variables for which an OR could be estimated, *p*-values were obtained from the Wald test of the coefficient of the regression model.

For the remaining categorical variables, *p*-values were obtained using Pearson’s chi-square test or Fisher’s exact test, as appropriate, depending on the number of observations in the categories.

Continuous variables were summarized using means and standard deviations or medians and interquartile ranges, as appropriate, and compared using the Student’s t-test or non-parametric tests, as appropriate. Imputation of missing values was not performed and all analyses were carried out using only available cases for each variable.

Survival outcomes, including overall survival (OS, defined as time from the date of diagnosis to death from any cause), disease-free survival (DFS, defined as the time from the date of curative-intent surgery to the first occurrence of disease recurrence or death from any cause), and progression-free survival (PFS, defined as time from the start of treatment until the first documented event of disease progression or death from any cause) for first- and second-line systemic therapies, were investigated using the Kaplan–Meier method to estimate median survival times and corresponding 95% confidence intervals (CIs).

Survival distributions between the two age cohorts were compared using the log-rank test.

To account for the substantial temporal heterogeneity of the study period, calendar year was included as a covariate in the multivariable Cox proportional hazards regression models, together with age group, sex, sidedness, stage, and number of sites of disease at diagnosis.

The proportional hazard assumption was checked using Schoenfeld residuals and no relevant deviation was detected.

Estimated adjusted hazard ratios (HRs) were reported with 95% CIs and *p*-values.

All statistical tests were two-sided, and a *p*-value < 0.05 was considered statistically significant. Given the exploratory nature of the study, no multiple testing correction was applied, and nominal *p*-values were reported. All statistical analyses were carried out using R version 4.3.2 (The R Foundation for Statistical Computing).

For each analysis, patients with missing values for the variables required for that specific analysis were excluded, and no imputation of missing data was performed. The proportion of missing data was reported for relevant variables.

## 3. Results

### 3.1. Patient Demographics and Baseline Characteristics

The study included a total of 686 patients, divided into two age-based cohorts: 153 young patients (Group A, ≤50 years) and 533 elderly patients (Group B, ≥75 years).

Regarding the calendar period of diagnosis, among younger patients, 30 (19.6%) were diagnosed between 1990 and 2004, 60 (39.2%) between 2005 and 2014, and 63 (41.2%) between 2015 and 2025. Among elderly patients, 160 (30.0%), 181 (34.0%), and 192 (36.0%) were diagnosed during the same periods, respectively.

No statistically significant differences were observed between the two groups regarding sex distribution (males: 55.6% in Group A vs. 55.5% in Group B) or common risk factors, in particular smoking, alcohol consumption, obesity and family history.

**Figure 2:**
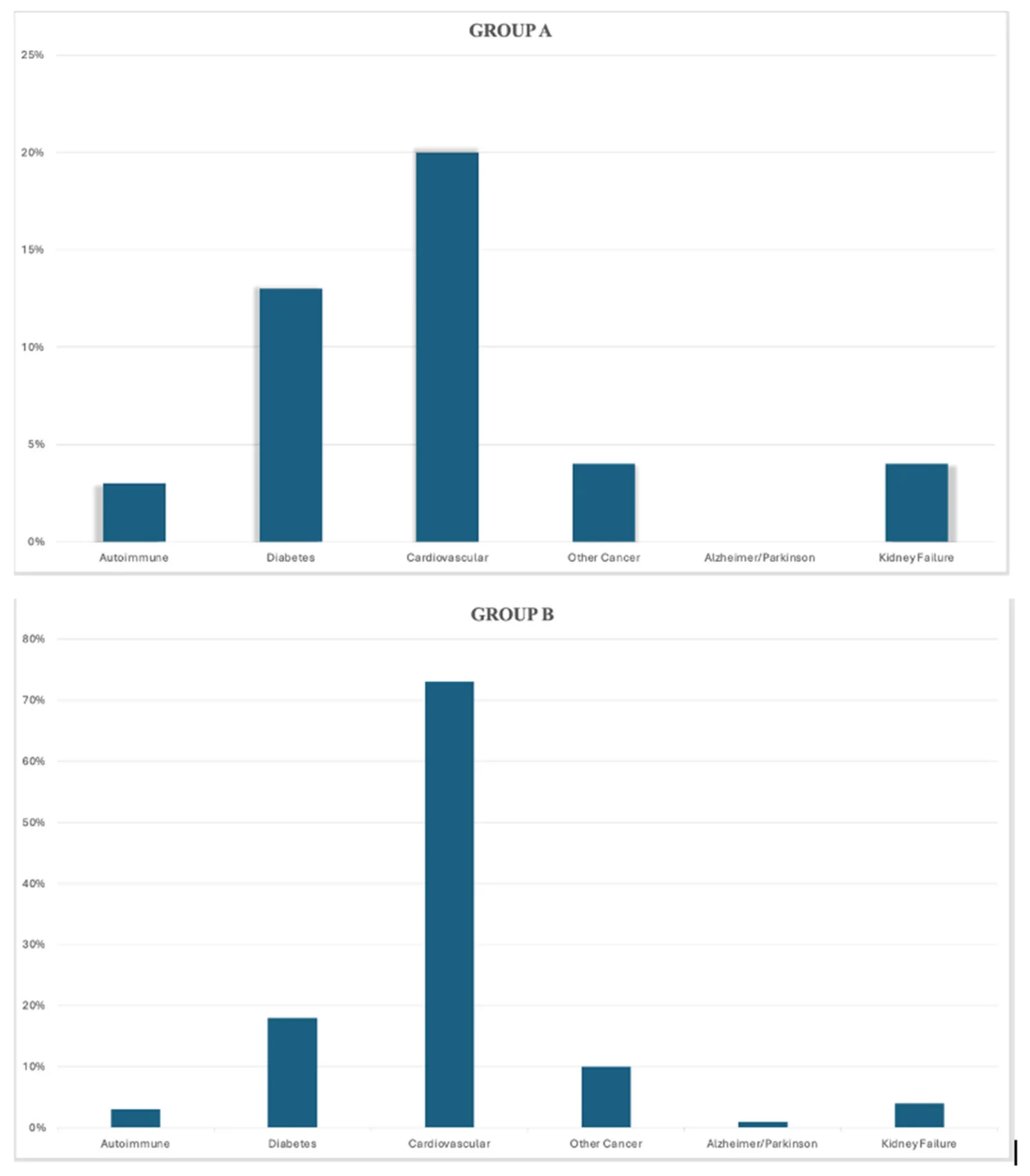
Comorbidities in younger and elderly patients (Group A and B respectively).

Among the comorbidities analyzed, no significant differences were observed between the two groups, except for cardiovascular diseases, which were markedly more prevalent in Group B (72% vs. 20%, OR 0.09 for Group A vs. Group B, 95% CI 0.05–0.18, *p* < 0.001) (Figure 2).

Further details can be found in the Supplementary Table.

### 3.2. Clinico-Pathological Features at Diagnosis

At diagnosis, Group A exhibited a higher prevalence of clinical and pathological features associated with adverse disease compared with Group B.

Group A showed a significantly higher incidence of Stage IV disease (66.90% vs. 47.1%; *p* < 0.001), T4 tumors (37.7% vs. 27.4%; *p* = 0.013) and N2 lymph node involvement (45.7% vs. 23.9%; *p* < 0.001).

Regarding anatomical location, distal (left-sided) tumors were more common in Group A (69.2% vs. 56.9%; *p* = 0.007), whereas proximal (right-sided) tumors were more frequent in Group B (36.6% vs. 22.6%).

Symptoms also differed significantly, likely attributable to the difference in tumor location between the two groups: Group A, in fact, was more likely to present with abdominal pain (25% vs. 12%, OR 2.59, 95% CI 1.22–5.52, *p* = 0.030) and altered bowel habits (32% vs. 18.4%, OR 2.17, 95% CI 1.09–4.35, *p* = 0.040), while diagnosis in Group B was more often associated with anemia (25.7% vs. 3%, OR 0.09 for Group A vs. Group B, 95% CI 0.02–0.38, *p* = 0.001).

Detailed pathological variables, including tumour budding and vascular invasion, were available only for a limited subset of patients and were therefore interpreted as exploratory findings because of substantial missingness.

Among patients with available pathological data, no statistically significant differences between the two age groups were observed in terms of histological grading, peritumoral deposits, or neural invasion.

Tumour budding, assessed according to the ITBCC 2016 criteria, was more frequently observed in Group B than in Group A (73.7% vs. 50.0%; OR 6.40, 95% CI 1.35–30.37; *p* = 0.019; evaluable patients: 50/686). Conversely, vascular invasion was more prevalent in Group A than in Group B (82.9% vs. 64.2%; OR 3.97, 95% CI 1.23–12.89; *p* = 0.022; evaluable patients: 88/686). These findings should be interpreted with considerable caution, as pathological variables were available for only a small and potentially selected subset of the overall cohort, which may introduce selection bias and substantially limit the generalizability of these comparisons. In addition, no central pathological review was performed. The limited availability of these pathological variables likely reflects the long study period (1990–2025) and the evolution of pathological reporting practices over time.

A summary of the main characteristics is shown in Table 1.

### 3.3. Metastatic Patterns and Molecular Profile

The analysis of metastatic patterns and timing revealed significant differences between the two age cohorts. Among patients with metastatic disease, Group A had a significantly higher prevalence of synchronous metastatic disease, defined as metastases present at the time of CRC diagnosis or diagnosed within 6 months thereafter, compared with Group B (87.5% [98 patients] vs. 70.7% [253 patients], respectively; OR 0.34 for Group B vs. Group A, 95% CI 0.19–0.63, *p* = 0.001). Metachronous metastatic disease, defined as metastases diagnosed more than 6 months after the initial CRC diagnosis, was observed in 14/112 (12.5%) patients in Group A and 105/358 (29.3%) patients in Group B. Further details can be found in the Supplementary Table.

Regarding the specific sites of involvement at the moment of the diagnosis, a marked contrast was observed in peritoneal and hepatic spread; Group A showed a significantly higher predisposition for the peritoneum (16.3% vs. 8%, OR 0.42 Group B vs. Group A, 95% CI 0.25–0.71, *p* = 0.002) and liver (47.1% vs. 31%; OR 0.50 Group B vs. Group A, 95% CI 0.35–0.73, *p* < 0.001). While lymph node involvement appeared more frequent in Group A, these differences did not reach statistical significance. Finally, no age-related differences were found for lung metastases.

Among patients with available molecular data, exploratory molecular analysis suggested differences between the two groups. NRAS mutations were significantly less common in Group A than in Group B (1.5% vs. 13.8%; OR 10.88 for Group B vs. Group A, 95% CI 1.16–102.07, *p* = 0.037; n = 69 and n = 29, respectively). No BRAF V600E mutations were detected in Group A compared with a 23.3% mutation rate in Group B (*p* < 0.001; n = 69 and n = 30, respectively). A trend toward a higher prevalence of microsatellite stability (pMMR) was also observed in Group A (86.2% vs. 75.5%; *p* = 0.079; n = 87 and n = 86, respectively). No significant difference was observed in KRAS mutation distribution between the two groups (n = 69 and n = 30, respectively). However, these differences should be interpreted with caution, as molecular testing was not uniformly available throughout the study period and may have been influenced by calendar era and clinical setting.

### 3.4. Treatment Patterns

Therapeutic approaches were heavily influenced by age. In the adjuvant setting, Group A was significantly more likely to receive chemotherapy (56.5% [39/69] vs. 11.4% [31/271]; OR 0.10 Group B vs. Group A, 95% CI 0.05–0.18, *p* < 0.001), predominantly with doublet regimens like XELOX (58.3%) or FOLFOX (27.8%). Conversely, Group B received mostly monotherapy with De Gramont (35.5%) or Capecitabine (32.2%). The 39 young patients and 31 elderly patients who received adjuvant treatment were distributed as follows: in Group A, 9 patients at stage II, 23 at stage III, and 7 at stage IV following resection of the primary tumour and metastasectomy; in Group B, 7 patients at stage II, 20 at stage III, and 4 at stage IV. Adjuvant chemotherapy administration was evaluated among patients with available systemic treatment information who underwent curative-intent surgical resection for stage II–III CRC, as well as stage IV patients undergoing complete resection of the primary tumor and metastasectomy. Because of the retrospective design and the long study period, formal criteria for adjuvant chemotherapy eligibility were not consistently documented and could not be reliably reconstructed for all patients. Therefore, the analysis was based on treatment information availability rather than on a retrospectively defined eligibility population.

In the metastatic setting, defined as stage IV disease at diagnosis or disease recurrence (comprising 120 patients in Group A and 378 patients in Group B, including both distant metastases and locally advanced inoperable recurrences), first-line systemic treatment was administered to 80.0% (96/120) of patients in Group A compared with 36.5% (138/378) of patients in Group B (OR 0.14, 95% CI 0.09–0.24, *p* < 0.001), whereas the remaining patients (20.0% [24/120] in Group A and 63.5% [240/378] in Group B) did not receive any systemic therapy. Group B was also significantly less likely to receive subsequent lines of therapy: second-line treatment was administered to 60.0% (72/120) of patients in Group A and 10.8% (41/378) in Group B (OR 0.08, 95% CI 0.05–0.13, *p* < 0.001); third-line treatment to 30.8% (37/120) and 4.5% (17/378), respectively (OR 0.11, 95% CI 0.06–0.20, *p* < 0.001); and fourth-line treatment to 14.2% (17/120) and 1.3% (5/378), respectively (OR 0.08, 95% CI 0.03–0.23, *p* < 0.001).

In both groups, first- and second-line systemic treatments were mainly based on fluoropyrimidines, oxaliplatin, and irinotecan, including doublet regimens (FOLFOX, XELOX, FOLFIRI, XELIRI), triplet regimens (FOLFOXIRI), and monotherapy (capecitabine and 5-fluorouracil), administered either alone or in combination with anti-EGFR agents (panitumumab or cetuximab) or antiangiogenic agents (bevacizumab or aflibercept). One patient in Group A received nivolumab plus ipilimumab in the first line. In the third-line setting, treatment options included the aforementioned doublet regimens or monotherapies, as well as trifluridine/tipiracil, with or without bevacizumab, available since 2017, and regorafenib, available since 2015. In the fourth-line setting, the aforementioned treatments were also used, with the addition of fruquintinib, available since 2024.

Further details can be found in the Supplementary Table.

### 3.5. Survival Outcomes

Group A achieved superior long-term survival outcomes. The median OS was 52.8 months for Group A compared to 26.4 months for Group B (adjusted HR 2.29, 95% CI 1.57–3.54, *p* < 0.001). Similarly, among patients who underwent curative-intent surgery, DFS was significantly longer in Group A (median survival time: 28.6 vs. 19.1 months; adjusted HR 2.26, 95% CI 1.55–3.29, *p* < 0.001) (Table 2 and Table 3).

**Figure 3:**
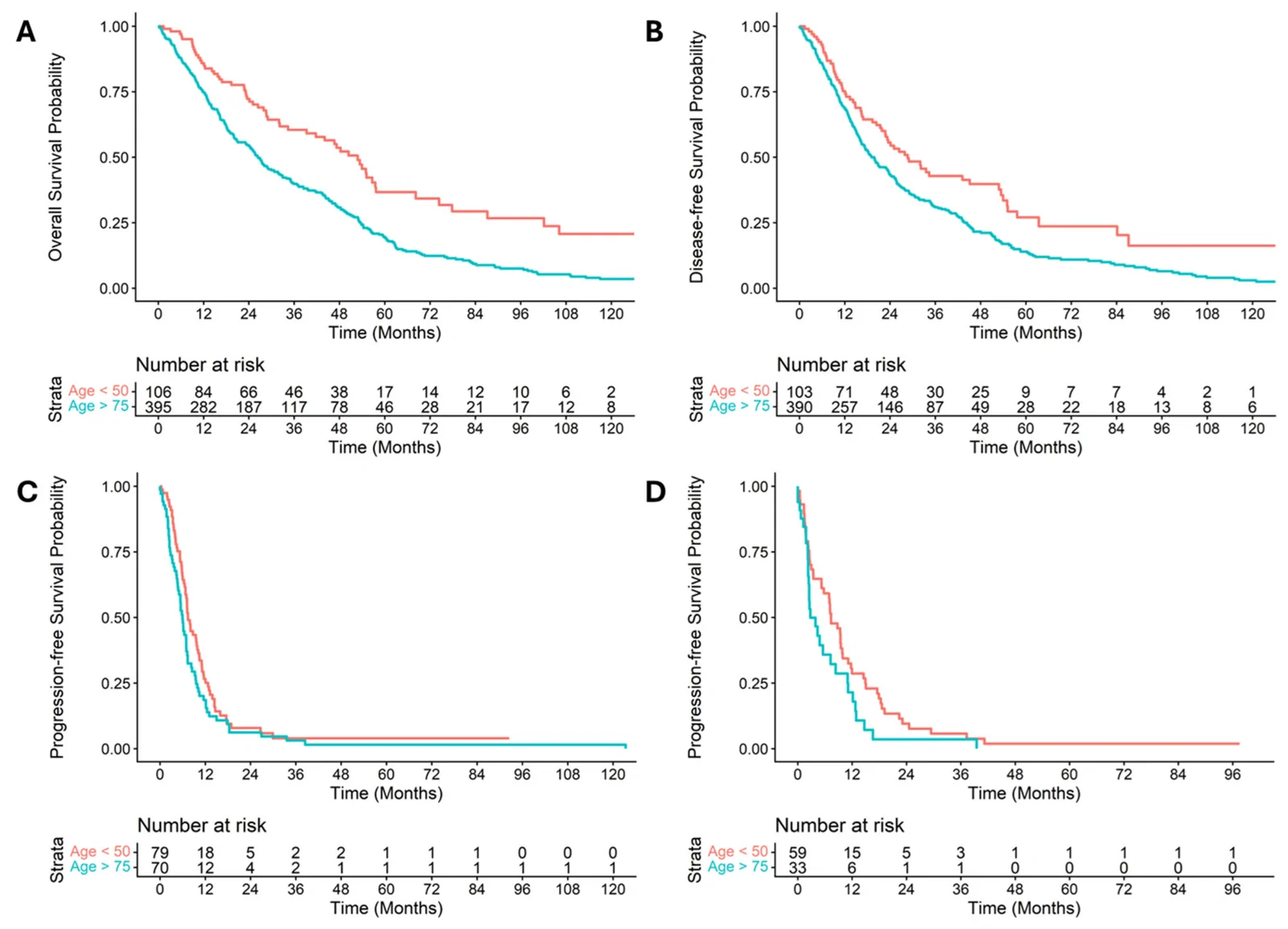
Kaplan–Meier curves for overall survival (**A**), disease-free survival (**B**), progression-free survival in the first- (**C**) and second-line (**D**) metastatic settings according to age cohort.

Regarding PFS in the metastatic setting, no statistically significant differences were found, either for first-line (median 7.4 vs. 6.0 months; adjusted HR 0.72, 95% CI 0.35–1.51, *p* = 0.38) or for second-line therapy (median 7.3 vs. 2.8 months; adjusted HR 1.18, 95% CI 0.47–2.99, *p* = 0.72) (Figure 3).

Finally, the overall recurrence rate was significantly higher in Group B (72.2% vs. 56.4%; OR 2.00 Group B vs. Group A, 95% CI 1.12–3.57, *p* = 0.018). Among the sites of recurrence analysed (liver, lungs, lymph nodes, bone, central nervous system, local), Group B had a lower incidence of peritoneal recurrence than Group A (12% vs. 19.2%, OR 0.42, 95% CI 0.25–0.71, *p* = 0.002).

## 4. Discussion

The present study provides a comprehensive comparison between younger and elderly patients with colorectal cancer, highlighting clinically meaningful differences between the two populations. Although baseline demographic variables and traditional risk factors were largely comparable, the two cohorts diverged substantially in terms of disease presentation, metastatic behavior, treatment patterns and survival outcomes.

First, our findings support the observation that CRC in young patients presents with a more adverse clinicopathological profile at diagnosis. A significantly higher proportion of young individuals were diagnosed with stage IV disease, T4 tumors and N2 nodal involvement. These data are consistent with several reports describing younger patients with a more advanced disease and adverse clinicopathological features [12,13,14,19,20,21,22,23].

The higher frequency of advanced disease observed among younger patients may reflect delayed diagnosis, but alternative explanations should also be considered. Differences in tumor location, exposure to screening programs, healthcare utilization and mechanisms of disease detection may have contributed to the observed stage distribution. Therefore, the more advanced presentation observed in younger patients should not be attributed solely to diagnostic delay.

The anatomical distribution further supports this interpretation. Young patients more frequently show distal (left-sided) tumors, whereas proximal tumors predominated in the elderly patients. This difference in localization plausibly explains the distinct symptomatic patterns observed: younger individuals more often presented with abdominal pain and altered bowel habits, whereas elderly patients were more frequently diagnosed because of anemia or through screening procedures. Distal tumors tend to become symptomatic earlier due to luminal narrowing and obstructive phenomena; however, in younger individuals, these symptoms may not immediately prompt endoscopic evaluation. Consequently, despite being potentially symptomatic, diagnosis may still occur late.

In the metastatic setting, young patients displayed a higher rate of synchronous disease and a greater predisposition to peritoneal and hepatic involvement. These findings may reflect differences in metastatic patterns, although the underlying biological mechanisms cannot be established from this retrospective analysis.

From a molecular standpoint, our data contribute to an ongoing and somewhat controversial debate. The literature on molecular differences between early- and late-onset CRC remains heterogeneous and, at times, contradictory. Some series have reported higher rates of MSI-high tumors and hereditary syndromes in younger populations, while others describe predominantly microsatellite-stable (pMMR) disease [21,24,25,26,27,28,29,30,31].

In our cohort, younger patients showed a trend toward higher microsatellite stability and an absence of BRAF V600E mutations, which were instead more frequent in the elderly group. Furthermore, NRAS mutations were significantly less common in younger individuals. However, these differences should be interpreted with caution, as molecular testing was not uniformly available throughout the study period and may have been influenced by calendar era and clinical setting. Therefore, the interpretation of molecular findings is limited by the substantial proportion of missing molecular data and by the evolution of molecular testing practices over the study period.

One of the most important aspects of our results concerns treatment patterns and outcomes. Young patients were significantly more likely to receive adjuvant chemotherapy and intensive doublet regimens. In the metastatic setting, they were treated with more lines of therapy and more intensive treatment combinations. However, the lower proportion of elderly patients receiving subsequent treatment lines should be interpreted with caution, as receipt of later-line therapy is conditional on survival, disease progression, clinical fitness and continued eligibility for treatment.

Long-term outcomes further clarify this dynamic. Overall survival and disease-free survival were significantly superior in the younger cohort, despite their higher initial disease burden. This survival advantage may reflect better performance status, fewer competing comorbidities, greater tolerance to intensive regimens and access to multiple lines of therapy. In contrast, elderly patients, despite sometimes presenting with less adverse pathological markers, were less frequently treated with combination chemotherapy and rarely reached later lines of treatment, reflecting both clinical prudence and frailty considerations.

Furthermore, our analysis revealed no significant differences in Progression-Free Survival between the two cohorts, including the second-line setting. This finding suggests that, despite potential distinct clinical features at presentation, younger and elderly patients derive a comparable benefit from standard therapeutic sequencing.

This study has several important strengths. First, it is based on a large institutional database comprising 2345 patients with colorectal cancer managed over a prolonged period at a tertiary referral cancer center, providing real-world evidence that reflects routine clinical practice. By comparing two extreme age cohorts (≤50 vs. ≥75 years), we minimized the potential confounding effect of intermediate age groups and more clearly highlighted the clinicopathological, molecular and therapeutic differences between younger and elderly patients with colorectal cancer.

Furthermore, unlike many previous studies focusing primarily on epidemiological trends or survival outcomes, our analysis provides a comprehensive characterization of these two populations by simultaneously integrating clinical presentation, pathological features, molecular profile, treatment patterns and long-term survival.

However, some limitations must be acknowledged. First, detailed treatment data, such as exact chemotherapy dosages, dose reductions, and adherence, were not consistently available, limiting our ability to fully evaluate the impact of dose intensity on survival outcomes. Similarly, survival analyses were constrained by missing survival times, which reduced the evaluable sample size. Moreover, the substantial missingness observed for some variables represents a limitation of the study, as missing data may be related to treatment era or patient selection.

As this was a single-center study conducted at a tertiary referral cancer center, referral and selection bias cannot be excluded. Referral patterns were not systematically captured in the retrospective database and therefore the generalizability of our findings to the broader CRC population should be considered with caution.

The selection of two extreme age groups (≤50 and ≥75 years), with exclusion of patients aged 51–74 years, was intended to maximize the contrast between younger and elderly patients. However, this design may limit the generalizability of the findings to the broader CRC population and does not eliminate potential confounding related to differences in clinical characteristics, treatment patterns, and calendar period.

Moreover, multivariable analyses were performed for covariates with complete data. However, information on all potentially prognostic patient, tumor, and treatment-related factors was not available and residual confounding is likely to affect the results of the analyses. Data on the specific cause of death were also unavailable, precluding competing-risk analyses and limiting the assessment of cancer-specific mortality.

Finally, the long observational period (1990–2025) represents an important source of temporal heterogeneity. During this period, diagnostic procedures, molecular characterization, surgery, systemic and targeted therapies and patient management evolved substantially. These changes may have influenced treatment patterns and survival outcomes and represent an additional source of potential confounding. However, the multivariable models were adjusted for calendar year to mitigate this effect. Finally, the large number of secondary and exploratory comparisons increases the possibility of type I error. The presented findings should therefore be considered hypothesis-generating and require validation.

## 5. Conclusions

In conclusion, our findings indicate that CRC in younger individuals is characterized by several features associated with more adverse clinicopathological features, including more frequent presentation at advanced stages and distinct metastatic and molecular characteristics. Younger patients also showed longer survival in our cohort; however, this finding should be interpreted cautiously, as differences in treatment exposure, baseline fitness and other unmeasured or incompletely captured factors may have contributed to the observed outcomes.

## Figures and Tables

**Table 1:** Main characteristics of the population analyzed. For variables for which an odds ratio (OR) could be estimated, *p*-values were obtained from the Wald test of the coefficient of a logistic regression model. For the remaining categorical variables, *p*-values were obtained using Pearson’s chi-square test or Fisher’s exact test, as appropriate based on the number of observations in the categories.

		Overall (N = 686)		≤ 50 yo (N = 153)		≥75 yo (N = 533)					
	Category	Number	%	Number	%	Number	%	OR	95% CI		*p*-Value
Gender	Female	305	44.44	68	44.37	237	44.47				
	Male	381	55.56	85	55.63	296	55.53	1.00	0.69	1.43	0.984
	N. missing	0		0		0					
Sidedness	Left	389	59.66	101	69.18	288	56.92				0.007
	Right	218	33.44	33	22.60	185	36.56				
	Transverse	45	6.90	12	8.22	33	6.52				
	N. missing	34		7		27					
T	X	21	3.98	0	0.00	21	5.08				0.013
	1	15	2.85	4	3.51	11	2.66				
	2	32	6.07	4	3.51	28	6.78				
	3	303	57.50	63	55.26	240	58.11				
	4	156	29.60	43	37.72	113	27.36				
	N. missing	159		39		120					
N	0	220	41.43	27	23.28	193	46.51				<0.001
	1	159	29.94	36	31.03	123	29.64				
	2	152	28.63	53	45.69	99	23.86				
	N. missing	155		37		118					
Stage	1	34	5.40	6	4.23	28	5.73				<0.001
	2	119	18.89	13	9.15	106	21.72				
	3	152	24.12	28	19.72	124	25.41				
	4	325	51.59	95	66.90	230	47.14				
	N. missing	56		11		45					
Grading	1	30	6.28	7	7.29	23	6.21				0.526
	2	301	62.97	54	56.25	247	64.66				
	3	147	30.75	35	36.46	111	29.13				
	N. missing	208		57		152					
Metachronous Synchronous	Metachronous	119	25.32	14	12.50	105	29.33				
	Synchronous	351	74.68	98	87.50	253	70.67	0.34	0.19	0.63	0.001
Surgery	Yes	537	84.43	119	85.00	418	84.27				
	No	99	15.57	21	15.00	78	15.73	1.06	0.63	1.78	0.834
Recurrence	Yes	296	70.14	31	56.36	265	72.21				
	No	126	29.86	24	43.64	102	27.79	0.50	0.28	0.89	0.018

**Table 2:** Survival outcomes according to age group; median OS was estimated with Kaplan Meier method. Abbreviations: OS = overall survival; DFS = disease-free survival; PFS = progression-free survival; IL = first-line treatment; IIL= second-line treatment; CI = confidence interval.

		N Patients	N Events	Median Survival Time (Months)	95% CI	
**OS**	Group A	106	58	52.8	39.3	57.7
	Group B	395	315	26.4	23.7	30.7
**DFS**	Group A	103	63	28.6	22.7	53.4
	Group B	390	317	19.1	16.9	23.6
**PFS IL**	Group A	68	19	7.4	6.7	10.1
	Group B	70	66	6.0	4.8	7.2
**PFS IIL**	Group A	59	53	7.3	5.3	9.9
	Group B	33	30	2.8	2.4	11.0

**Table 3:** Univariable and multivariable Cox proportional hazards regression analyses of survival outcomes. Abbreviations: OS = overall survival; DFS = disease -free survival; PFS = progression-free survival; IL = first-line treatment; IIL= second-line treatment; HR = hazard ratio; CI = confidence interval; Ref. = reference group.

			Univariable Analysis			Multivariable Analysis	
		HR	95% CI	*p*-Value	HR	95% CI	*p*-Value
**OS**	Group A Group B	Ref. 1.93	1.45–2.55	<0.001	Ref. 2.29	1.57–3.54	<0.001
**DFS**	Group A Group B	Ref. 1.56	1.19–2.05	0.001	Ref. 2.26	1.55–3.29	<0.001
**PFS IL**	Group A Group B	Ref. 1.36	0.97–1.92	0.074	Ref. 0.72	0.35–1.51	0.38
**PFS IIL**	Group A Group B	Ref. 1.57	0.99–2.47	0.055	Ref. 1.18	0.47–2.99	0.72

## Data Availability

The data presented in this study are available on request from the corresponding authors. The data are not publicly available due to privacy and ethical restrictions regarding patient medical records.

## Supplementary Materials

The following supporting information can be downloaded at: https://www.mdpi.com/article/10.3390/cancers18183063/s1, Table S1. Main characteristics of the population analyzed. For variables for which an odds ratio (OR) could be estimated, *p*-values were obtained from the Wald test of the coefficient of a logistic regression model. For the remaining categorical variables, *p*-values were obtained using Pearson’s chi-square test or Fisher’s exact test, as appropriate based on the number of observations in the categories.
