# Supplementary material for: Colorectal Cancer in Younger and Elderly Patients: A Real-World Analysis from the Modena Cancer Center
Source: Cancers (Basel). 2026 Sep 21;18(18):3063. doi: 10.3390/cancers18183063 (PMC13605757; doi:10.3390/cancers18183063)
Supplement: Supplementary file 1 [file cancers-18-03063-s001.zip › cancers-4575117-supplementary.pdf]

Table. S1 Main characteristics of the population analyzed. For variables for which an odds ratio (OR) could be estimated, p-values were obtained from the Wald test of the coefficient of a logistic regression model. For the remaining categorical variables, p-values were obtained using Pearson's chi-square test or Fisher's exact test, as appropriate based on the number of observations in the categories.

Variable	Details	Overall (N = 686) n (%)	<=50 anni (N = 153) n (%)	>=75 anni (N = 533) n (%)	>=75 anni vs <=50 anni p-value
Gender (F/M 0/1)	0	305 (44,44%)	68 (44,37%)	237 (44,47%)	
	1	381 (55,56%)	85 (55,63%)	296 (55,53%)	0,984
	N.missing	0	0	0	
Age	mean (SD)	73,83 (18,90)	41,38 (12,59)	83,14 (4,99)	<0,001
	N.missing	0	0	0	
Familiarity 0 yes/1no	0	38 (55,88%)	21 (55,26%)	17 (56,67%)	0,908
	1	30 (44,12%)	17 (44,74%)	13 (43,33%)	0,908
	N.missing	618	115	503	
Smoke-alcol-obesity 0 yes/1 no	0	29 (42,65%)	16 (42,11%)	13 (43,33%)	0,919
	1	39 (57,35%)	22 (57,89%)	17 (56,67%)	0,919
	N.missing	618	115	503	
Lynch sd-polyposis -history of CRC 0 yes/1no	0	10 (14,71%)	6 (15,79%)	4 (13,33%)	0,777
	1	58 (85,29%)	32 (84,21%)	26 (86,67%)	0,777
	N.missing	618	115	503	
Alteration bowel habits 0 yes/1no	0	42 (23,6%)	22 (31,88%)	20 (18,35%)	0,040
	1	136 (76,4%)	47 (68,12%)	89 (81,65%)	0,040
	N.missing	508	84	424	
Pain 0 yes/1no	0	30 (16,85%)	17 (24,64%)	13 (11,93%)	0,030
	1	148 (83,15%)	52 (75,36%)	96 (88,07%)	0,030
	N.missing	508	84	424	
Screening 0 yes/1no	0	17 (9,55%)	7 (10,14%)	10 (9,17%)	
	1	161 (90,45%)	62 (89,86%)	99 (90,83%)	0,830
	N.missing	508	84	424	
Perforation-occlusion 0 yes/1 no	0	24 (13,48%)	10 (14,49%)	14 (12,84%)	
	1	154 (86,52%)	59 (85,51%)	95 (87,16%)	0,754
	N.missing	508	84	424	
Weight loss 0 yes /1 no	0	15 (8,43%)	3 (4,35%)	12 (11,01%)	
	1	163 (91,57%)	66 (95,65%)	97 (88,99%)	0,132
	N.missing	508	84	424	
Anemia 0 yes/1 no	0	30 (16,85%)	2 (2,9%)	28 (25,69%)	
	1	148 (83,15%)	67 (97,1%)	81 (74,31%)	0,001
	N.missing	508	84	424	
Vomiting-nausea- loss of appetite 0yes/1 no	0	8 (4,49%)	2 (2,9%)	6 (5,5%)	
	1	170 (95,51%)	67 (97,1%)	103 (94,5%)	0,421
	N.missing	508	84	424	
Cardiovascular 0 yes/1 no	0	129 (55,6%)	15 (20%)	114 (72,61%)	

	1	103 (44,4%)	60 (80%)	43 (27,39%)	<0,001
	N.missing	454	78	376	
Diabetes 0 yes/1no	0	39 (16,81%)	10 (13,33%)	29 (18,47%)	
	1	193 (83,19%)	65 (86,67%)	128 (81,53%)	0,330
	N.missing	454	78	376	
Autoimmune 0 yes/1no	0	11 (4,74%)	2 (2,67%)	9 (5,73%)	
	1	221 (95,26%)	73 (97,33%)	148 (94,27%)	0,316
	N.missing	454	78	376	
Alzheimer/parkinson 0 yes/1no	0	2 (0,86%)	0 (0%)	2 (1,27%)	1,000
	1	230 (99,14%)	75 (100%)	155 (98,73%)	
	N.missing	454	78	376	
Previous cancer 0 yes/1no	0	19 (8,19%)	3 (4%)	16 (10,19%)	
	1	213 (91,81%)	72 (96%)	141 (89,81%)	0,121
	N.missing	454	78	376	
Chronic kidney failure 0 yes/1no	0	12 (5,17%)	3 (4%)	9 (5,73%)	
	1	220 (94,83%)	72 (96%)	148 (94,27%)	0,579
	N.missing	454	78	376	
Left/right/transverse 0/1/2	0	389 (59,66%)	101 (69,18%)	288 (56,92%)	0,007
	1	218 (33,44%)	33 (22,6%)	185 (36,56%)	
	2	45 (6,9%)	12 (8,22%)	33 (6,52%)	
	N. missing	34	7	27	
T	x	21 (3,98%)	0 (0%)	21 (5,08%)	0,013
T	1	15 (2,85%)	4 (3,51%)	11 (2,66%)	
T	2	32 (6,07%)	4 (3,51%)	28 (6,78%)	
T	3	303 (57,5%)	63 (55,26%)	240 (58,11%)	
T	4	156 (29,6%)	43 (37,72%)	113 (27,36%)	
T	N. missing	159	39	120	
N	0	220 (41,43%)	27 (23,28%)	193 (46,51%)	<0,001
N	1	159 (29,94%)	36 (31,03%)	123 (29,64%)	
N	2	152 (28,63%)	53 (45,69%)	99 (23,86%)	
N	N. missing	155 (0%)	37 (0%)	118 (0%)	
Stage	0	3 (0,48%)	1 (0,7%)	2 (0,41%)	<0,001
	1	31 (4,92%)	5 (3,52%)	26 (5,33%)	
	2	119 (18,89%)	13 (9,15%)	106 (21,72%)	
	3	152 (24,13%)	28 (19,72%)	124 (25,41%)	
	4	325 (51,59%)	95 (66,9%)	230 (47,13%)	
	N. missing	56	11	45	
Grading	1	30 (6,28%)	7 (7,29%)	23 (6,21%)	0.526
	2	301 (62,97%)	54 (56,25%)	247 (64,66%)	
	3	147 (30,75%)	35 (36,46%)	111 (29,13%)	
	N. missing	208	57	152	
Tumour infiltrating lymphocytes 1 yes /no 0	0	12 (41,38%)	9 (81,82%)	3 (16,67%)	
	1	17 (58,62%)	2 (18,18%)	15 (83,33%)	0,002
	N.missing	657	142	515	
Peritumoral deposits 1 yes/0 no	0	7 (30,43%)	2 (20%)	5 (38,46%)	
	1	16 (69,57%)	8 (80%)	8 (61,54%)	0,347
	N.missing	663	143	520	
Budding 1 yes/no 0	0	16 (32%)	6 (50%)	10 (26,32%)	

	1	34 (68%)	6 (50%)	28 (73,68%)	0,019
	N.missing	636	141	495	
vascular invasion 0 yes/1 no	0	63 (71,59%)	29 (82,86%)	34 (64,15%)	
	1	25 (28,41%)	6 (17,14%)	19 (35,85%)	0,022
	N.missing	598	118	480	
neural invasion 0 yes/1 no	0	40 (60,61%)	11 (55%)	29 (63,04%)	
	1	26 (39,39%)	9 (45%)	17 (36,96%)	0,540
	N.missing	620	133	487	
KRAS 12-13 0 wt/1 mutated	0	75 (75%)	56 (80%)	19 (63,33%)	
	1	25 (25%)	14 (20%)	11 (36,67%)	0,082
	N.missing	586	83	503	
KRAS 0 wt/1 mutated	0	55 (56,12%)	39 (56,52%)	16 (55,17%)	
	1	43 (43,88%)	30 (43,48%)	13 (44,83%)	0,902
	N.missing	588	84	504	
NRAS 0 wt/1 mutated	0	93 (94,9%)	68 (98,55%)	25 (86,21%)	
	1	5 (5,1%)	1 (1,45%)	4 (13,79%)	0,037
	N.missing	588	84	504	
BRAF 0 wt/1 mutated	0	92 (92,93%)	69 (100%)	23 (76,67%)	<0,001
	1	7 (7,07%)	0 (0%)	7 (23,33%)	
	N.missing	587	84	503	
MMR.0 pMMR/1 dMMR	0	140 (80,92%)	75 (86,21%)	65 (75,58%)	
	1	33 (19,08%)	12 (13,79%)	21 (24,42%)	0,079
	N. missing	513 (0%)	66 (0%)	447 (0%)	
Metachronous Synchronous 0/1	0	119 (25,32%)	14 (12,5%)	105 (29,33%)	
	1	351 (74,68%)	98 (87,5%)	253 (70,67%)	0,001
	N.missing	216	41	175	
Number of metastatic sites at the time of diagnosis	1	276 (67,15%)	60 (60,61%)	216 (69,23%)	0,213
	2+	135 (32,85%)	39 (39,39%)	96 (30,77%)	
	N.missing	275	54	221	
Liver 0no/1 yes	0	449 (65,45%)	81 (52,94%)	368 (69,04%)	
	1	237 (34,55%)	72 (47,06%)	165 (30,96%)	<0,001
	N. missing	0	0	0	
Lungs 0no/1 yes	0	619 (90,36%)	137 (89,54%)	482 (90,6%)	
	1	66 (9,64%)	16 (10,46%)	50 (9,4%)	0,696
	N. missing	1	0	1	
Lymphnode 0no/1 yes	0	625 (91,24%)	134 (87,58%)	491 (92,29%)	
	1	60 (8,76%)	19 (12,42%)	41 (7,71%)	0,072
	N. missing	1	0 (0%)	1 (0%)	
Peritoneum 0no/1 yes	0	619 (90,36%)	128 (83,66%)	491 (92,29%)	
	1	66 (9,64%)	25 (16,34%)	41 (7,71%)	0,002
	N. missing	1	0	1	
Othes sites 0no/1 yes	0	651 (95,04%)	144 (94,12%)	507 (95,3%)	
	1	34 (4,96%)	9 (5,88%)	25 (4,7%)	0,553
	N. missing	1	0	1	
Surgery 0 yes/1 no	0	537 (84,43%)	119 (85%)	418 (84,27%)	
	1	99 (15,57%)	21 (15%)	78 (15,73%)	0,834
	N. missing	50	13	37	
Adjuvant treatment 1.yes/0.no	0	270 (79,41%)	30 (43,48%)	240 (88,56%)	

	1	70 (20,59%)	39 (56,52%)	31 (11,44%)	<0,001
	N. missing	346	84	262	
Recurrence 0 yes/1 no	0	296 (70,14%)	31 (56,36%)	265(72,21%)	
	1	126 (29,86%)	24 (43,64%)	102 (27,79%)	0,018
Lymphnode 0 yes/1 no	0	38 (19,9%)	8 (30,77%)	30 (18,18%)	
	1	153 (80,1%)	18 (69,23%)	135 (81,82%)	0,141
	N.missing	495	127	368	
Retroperitoneum 0 yes/1 no	0	6 (3,14%)	2 (7,69%)	4 (2,42%)	
	1	185 (96,86%)	24 (92,31%)	161 (97,58%)	0,175
	N.missing	495	127	368	
Liver	0	66 (34,55%)	7 (26,92%)	59 (35,76%)	
	1	125 (65,45%)	19 (73,08%)	106 (64,24%)	<0,001
	N.missing	495	127	368	
Local Recurrence 0 yes/1 no	0	38 (19,9%)	5 (19,23%)	33 (20%)	
	1	153 (80,1%)	21 (80,77%)	132 (80%)	0,927
	N.missing	495	127	368	
Lungs 0 yes/1 no	0	57 (29,84%)	7 (26,92%)	50 (30,3%)	
	1	134 (70,16%)	19 (73,08%)	115 (69,7%)	0,696
	N.missing	495	127	368	
Pancreas 0 yes/1 no	0	3 (1,57%)	1 (3,85%)	2 (1,21%)	
	1	188 (98,43%)	25 (96,15%)	163 (98,79%)	0,342
	N.missing	495	127	368	
Peritoneum 0 yes/1 no	0	25 (13,09%)	5 (19,23%)	20 (12,12%)	
	1	166 (86,91%)	21 (80,77%)	145 (87,88%)	0,002
	N.missing	495	127	368	
Fat tissue 0 yes/1 no	0	4 (2,09%)	1 (3,85%)	3 (1,82%)	
	1	187 (97,91%)	25 (96,15%)	162 (98,18%)	0,512
	N.missing	495	127	368	
Pelvis 0 yes/1 no	0	4 (2,09%)	0 (0%)	4 (2,42%)	1,000
	1	187 (97,91%)	26 (100%)	161 (97,58%)	
	N.missing	495	127	368	
Subcutaneous 0 yes/1 no	0	1 (0,52%)	0 (0%)	1 (0,61%)	1,000
	1	190 (99,48%)	26 (100%)	164 (99,39%)	
	N.missing	495	127	368	
Brain 0 yes/1 no	0	5 (2,62%)	0 (0%)	5 (3,03%)	1,000
	1	186 (97,38%)	26 (100%)	160 (96,97%)	
	N.missing	495	127	368	
Bone 0 yes/1 no	0	8 (4,19%)	1 (3,85%)	7 (4,24%)	
	1	183 (95,81%)	25 (96,15%)	158 (95,76%)	0,925
	N.missing	495	127	368	
